# A constructive non-local means algorithm for low-dose computed tomography denoising with morphological residual processing

**DOI:** 10.1371/journal.pone.0291911

**Published:** 2023-09-27

**Authors:** Dawa Chyophel Lepcha, Ayush Dogra, Bhawna Goyal, Vishal Goyal, Vinay Kukreja, Durga Prasad Bavirisetti

**Affiliations:** 1 Department of ECE, Chandigarh University, Mohali, Punjab, India; 2 Chitkara University Institute of Engineering and Technology, Chitkara University, Punjab, India; 3 Department of ECE and UCRD, Chandigarh University, Mohali, Punjab, India; 4 GLA University, Mathura, India; 5 Department of Computer Science, Norwegian University of Science and Technology, Trondheim, Norway; Islamia University of Bahawalpur: The Islamia University of Bahawalpur Pakistan, PAKISTAN

## Abstract

Low-dose computed tomography (LDCT) has attracted significant attention in the domain of medical imaging due to the inherent risks of normal-dose computed tomography (NDCT) based X-ray radiations to patients. However, reducing radiation dose in CT imaging produces noise and artifacts that degrade image quality and subsequently hinders medical disease diagnostic performance. In order to address these problems, this research article presents a competent low-dose computed tomography image denoising algorithm based on a constructive non-local means algorithm with morphological residual processing to achieve the task of removing noise from the LDCT images. We propose an innovative constructive non-local image filtering algorithm by means of applications in low-dose computed tomography technology. The nonlocal mean filter that was recently proposed was modified to construct our denoising algorithm. It constructs the discrete property of neighboring filtering to enable rapid vectorized and parallel implantation in contemporary shared memory computer platforms while simultaneously decreases computing complexity. Subsequently, the proposed method performs faster computation compared to a non-vectorized and serial implementation in terms of speed and scales linearly with image dimension. In addition, the morphological residual processing is employed for the purpose of edge-preserving image processing. It combines linear lowpass filtering with a nonlinear technique that enables the extraction of meaningful regions where edges could be preserved while removing residual artifacts from the images. Experimental results demonstrate that the proposed algorithm preserves more textural and structural features while reducing noise, enhances edges and significantly improves image quality more effectively. The proposed research article obtains better results both qualitatively and quantitively when compared to other comparative algorithms on publicly accessible datasets.

## 1. Introduction

Computed tomography (CT) is one of the most pivotal medical imaging technologies for clinical diagnosis where it uses X-ray radiation to generate cross-sectional images of the internal human body [1,2]. The internal abnormalities of the body for instance tumours, bone fractures, and vascular disorders, can be accurately and non-invasively detected using computed tomography (CT). It has been routinely utilised by clinicians in recent years to identify and monitor diseases like cancer, lung nodules, and internal injuries. Anatomical information with higher temporal spatial resolution can be obtained via CT scans and therefore several stakeholders get an advantage from CT images, particularly in pathological diagnosis and treatment. Widespread utilisation of CT is growing public concerns about its safety irrespective of the fact that it is extremely vital for disease diagnosis [3]. A significant concern about radiation exposure has arisen because CT images are produced by omitting X-ray beams at the body. In comparison to a standard X-ray, a CT scan exposes the patient to far more radiation during a single session. For instance, 10 days of background radiation are equal to the radiation a patient would get during a chest X-ray radiography. The radiation that a person is exposed to on a daily basis from cosmic and natural sources is known as background radiation. The radiation dose from a chest CT scan is comparable to two years of ambient radiation. Therefore, computed tomography carries a significantly higher radiation risk, particularly for those who have undergone several CT scans [4,5]. Radiation affects people of all ages, but children are more vulnerable than adults because of their developing bodies and longer lifespans which means more CT scans may be required. According to studies, children who receive cumulative doses from several head scans are up to three times more likely to develop disorders like leukaemia and brain tumours. Finding a solution to the radiation issue is crucial, especially in light of the benefits of CT scans for diagnosis. Using lower doses of X-ray current is one method for reducing the radiation risk [6,7].

The CT-related X-ray radiation has the potential to harm human health irreparably and sometimes even cause harmful diseases such as cancer. As a matter of fact, a widely recognized preposition in CT-based studies over the past few years has been to decrease the radiation dose in CT as low as reasonably achievable (ALARA) [8]. Medical problems such as metabolic anomalies, cancer, leukaemia and other ailments are often triggered by excessive X-ray radiation [9]. The problem of high radiation dose is currently the main barrier to the potential expansion of CT clinical applications. Thus, the ALARA concept [10] states that the radiation impact of X-rays on human beings should be as negligible as possible while ensuring image quality to achieve the diagnostic requirements which is widely encouraged around the world [11]. But decreasing radiation dose can increase multiplicative noise in projections that results in noise and irregular artifacts in CT images. The noise and artifacts that are inevitably introduced in the reconstructed images as a result of reducing radiation dose significantly hamper the diagnosis [12,13]. Also, compared to CT scans taken at normal doses, the ones that are obtained with low radiation doses are less sharp and precise. This means that they will not be reliable for diagnoses [14]. Due to the demand, there is significant research being done in the area of noise removal from low-dose CT (LDCT). To improve the calibre of the reconstructed CT images, numerous types of research have been carried out recently [15]. The researchers are consistently enthusiastic for achieving an image processing algorithm for competently suppressing noise and artifacts in the LDCT images. Nevertheless, it remains a challenging task due to its ill-posed nature. In recent times, numerous LDCT image-denoising techniques have been developed while achieving notable results [16,17].

In recent years, the dominant research emphasis on nonlocal means (NLM) that considers pixel similarity structure in the large-scale windows [18]. The non-local means theory is utilised in order to preserve edges and reduces noise adequately. The modified block-matching 3-dimsioonalal (*m-*BM3D*)* filtering method [19] integrates the benefits of spatial non-local means denoising and wavelet thresholding shrinking that reduces the complexness of *L*2 distance along with identical judgement by using hard threshold linear transformation. Trung et al. [20] presents a LDCT image denoising approach on the basis of image decomposition with sparse representation (IDSR), where it decomposes noisy image into three image components. However, noise in high and median components is effectively eliminated through manipulating the fact that the smaller image patches of noisy images could be considered as linear amalgamation of multiple components in the specified dictionary of denoised patches extracted from denoised images captured at almost similar region as the noisy images. For increasing the quality of LDCT images, a novel total variation (NTV) method is utilised [21]. The block matching 3D filter is used to generate fidelity term of the NTV method to effectively preserve details and edges since it executes well in edges and details preservation. To improve LDCT images, an efficient image denoising technique on the basis of discriminative weighted nuclear norm minimisation [22] is presented. This approach exploits the local entropy of the images for variable streak artifacts from tissue structure along with adaptively tuned WNNM weight components. In addition, a pre-processed images are also employed to increase block matching accuracy as well as the total variation method is used for decreasing residual artefacts in the reconstructed image even more. Traditional post-processing methods perform well in general, but they cannot eliminate the issue of over smoothing and could introduce new noises. Yuan *et al*. [23] recently proposes a method based on weighted coding approach on the basis of edge preserving median filtering and sparse non-local regularisation. This method shows great potential on removing noise and artefacts while reconstructing high image quality images.

Due to growth of deep learning in the domain of image processing in recent times. Deep learning-based research is also active in the domain of LDCT image denoising. Therefore, some deep learning-based LDCT image denoising algorithms are emerged in recent times [24–26]. Ren *et al*. [27] presented a deep learning strategy to alleviate some of the LDCT image denoising problems. In order to prevent the damage of shallow layer information while extracting rich feature details, this method utilizes dilated convolution rather than standard convolution to aggregate data from diverse receptive fields. For LDCT image denoising, a parallel-clone network (PCN) [28] is proposed that uses a modularised network design and exploits advantage of extraction of richer information. When compared to traditional models, this model preserves an identical or smaller amount of unknown network weights however could dramatically speed up the learning procedure. Pre-processing as well as post processing methods are incorporated into the dilated CNN to expand receptive fields in a novel CNN-based methods [29,30]. Chen et al. introduced residual learning for LDCT image denoising [29]. Moreover, skip connections were made available for network optimization. The efficiency of this method in preserving clarity and minimizing noise was then examined through meticulous experimental study. The FTV loss can preserve important structural information while reducing noise, resulting in CT scans of the highest caliber that are ready for radiologists to assess. A state-of-the-art intra-task knowledge transfer method was also proposed [30], which made use of the knowledge collected from NDCT images for training purposes. The CRM works to force the restored CT images distant from the LDCT samples while moving the NDCT samples in the latent space closer to the restored CT images.

Zhang et al. developed a task-oriented denoising network (TOD-Net) [31] along with task-oriented leverage data from downstream operations to enhance the family of LDCT denoising algorithms. According to the studies, task-oriented loss helps other task agnostic losses and enhances image quality in regions of interest that are task-based. Li et al. [32] introduce a denoising algorithm for improving image quality while reducing CT radiation exposure and the whole network structure is defined as having a top-down self-guiding strategy. Han et al. [24] propose a novel form of generative adversarial network (GAN) with dual-encoder and single-decoder strategy to enhance the capability of network to completely handle diverse types of information. In order to boost the effectiveness of feature extraction by strengthening features with self-similarity, the primary encoder channel of the generator is constructed. Further a novel technique is developed for eliminating image blur while training the convolutional neural network (CNN) denoiser with an activation map [25]. The focal region that the CNN classifier utilized to categorize the image is observed on the activation map. Activation map of an image is obtained utilizing trained CNN classifier which is trained to distinguish between CT images with lesions and those without lesions (a binary detection problem). The lesions and image edges are discovered to be activated in the activation map which highlights small features. Liu et al. [26] proposes a denoising method where it assumes that noise and irregular characteristics are connected and is used to generate DFSNE-Net, a network architecture that is sensitive to these features. MSC-DFPM discovers further abnormal features in this network, and SISA-FM transmits and filters them. Jiang et al. [33] introduced a denoising algorithm for improving image visual superiority of LDCT images. Residual learning supports network learning, and batch normalization counters performance decline caused on by adding additional neural network layers. The efficiency of the denoising algorithm and the generalizability of the model were evaluated using both real and simulated datasets. Trung et al. [34] developed a method based on a recently introduced dilated residual network for de-speckling of synthetic aperture radar. In order to enhance the receptive field and accelerate computation, pre- and post-processing layers are added. Utilising a GAN framework [35], Yin et al. [36] develops a novel denoising strategy. In recent years, a tuneable CycleGAN structure with a single generator for LDCT denoising was presented [37] and further a GAN with dual encoder and single decoder scheme is developed [38] for enhancing the networks potential to categorically deals with different types of image details. In the main encoder channel, a pyramid nonlocal attention module is constructed to enhance extraction of features efficiently.

Many GAN-based LDCT image denoising has been presented in recent times [39–41] and producing excellent results in case of both removal of noise and preserving structural information. Jiao et al. [39] presented a denoising algorithm to minimize noise in LDCT images. Li et al. [40] further constructed a technique for LDCT denoising based on the hybrid loss functions. The boundary regions might be highlighted by the adversarial loss. Recently a method based on artifacts sensing generative adversarial networks for LDCT image denoising was proposed [41]. First an artifacts direction sensing generator was designed. Based on the U-residual encoding and decoding structure in generator, an artifacts direction sensing sub-module (ADSS) was concatenated to improve the sensitivity of generator to artifacts direction features. Then the attention discriminator was designed for improving the potentiality of distinguishing noise and artifacts. The denoising performance of network was further improved by incorporating multiple loss functions.

This study presents a denoising scheme based on non-local means algorithm and morphological processing of its residuals in order to successfully reduce noise and artifacts in LDCT images. NL-means algorithm generally performs well in images with lots of repetitive structured patterns. By calculating a weighted average of the pixel values, it makes use of redundant information to lower noise. We avoid the sliding window design commonly used in neighbourhood filtering implementations in favour of more sophisticated concepts that enable faster implementation. To further improve noise suppression, we use lowpass filtering with morphological processing of residuals edge-preserving image processing algorithm. This can be accomplished by combining a linear lowpass filter with non-linear algorithms which enable the selection of significant portions of images where edges may be appropriately preserved. These regions are selected by morphologically processing the residuals of a linear filter in order to locate relevant regions with high amplitude edges and the optimal size. The original shape of the edges is recovered by integrating the meaningfully reconstructed regions with the output of the lowpass filter. This technique also enables you to adjust the contrast of final output image. The final output may be modified to meet the specific needs depending on the preference of parameters.

The main contributions of this study are:

1. This method proposes a competent LDCT image denoising method by combining the benefits of a constructive non-local means (NLM) algorithm and edge preserving processing where it adapts more state-of-the-art concepts and speed-ups processing time over other methods.
2. The method reduces the computational complexity of the prior non-local means (NLM) approach while presenting a fast vectorized and parallel implementation in contemporary shared memory computer platforms.
3. The edge preservation processing performs area opening and reconstruction on the low-pass linear filter residuals using morphological operators by avoiding blurring in the image regions.
4. The proposed method is able to achieves high-quality image reconstruction and further boosts the generalization for different types of noise properties resulting in better balance between visual perceptibility and quantitative evaluation.

The remainder part of this study is structured as follows. Section 2 illustrates the proposed LDCT image denoising framework based on a constructive non-local means algorithm with morphological residual processing in detail. The experimental details and results are presented using a publicly accessible dataset in Section 3 and the ablation study is illustrated in Section 4. Section 5 further elaborated inclusive discussion on results and Section 6 exhibits the conclusion and the future research scope.

## 2. Proposed image denoising method

**Fig 1:**
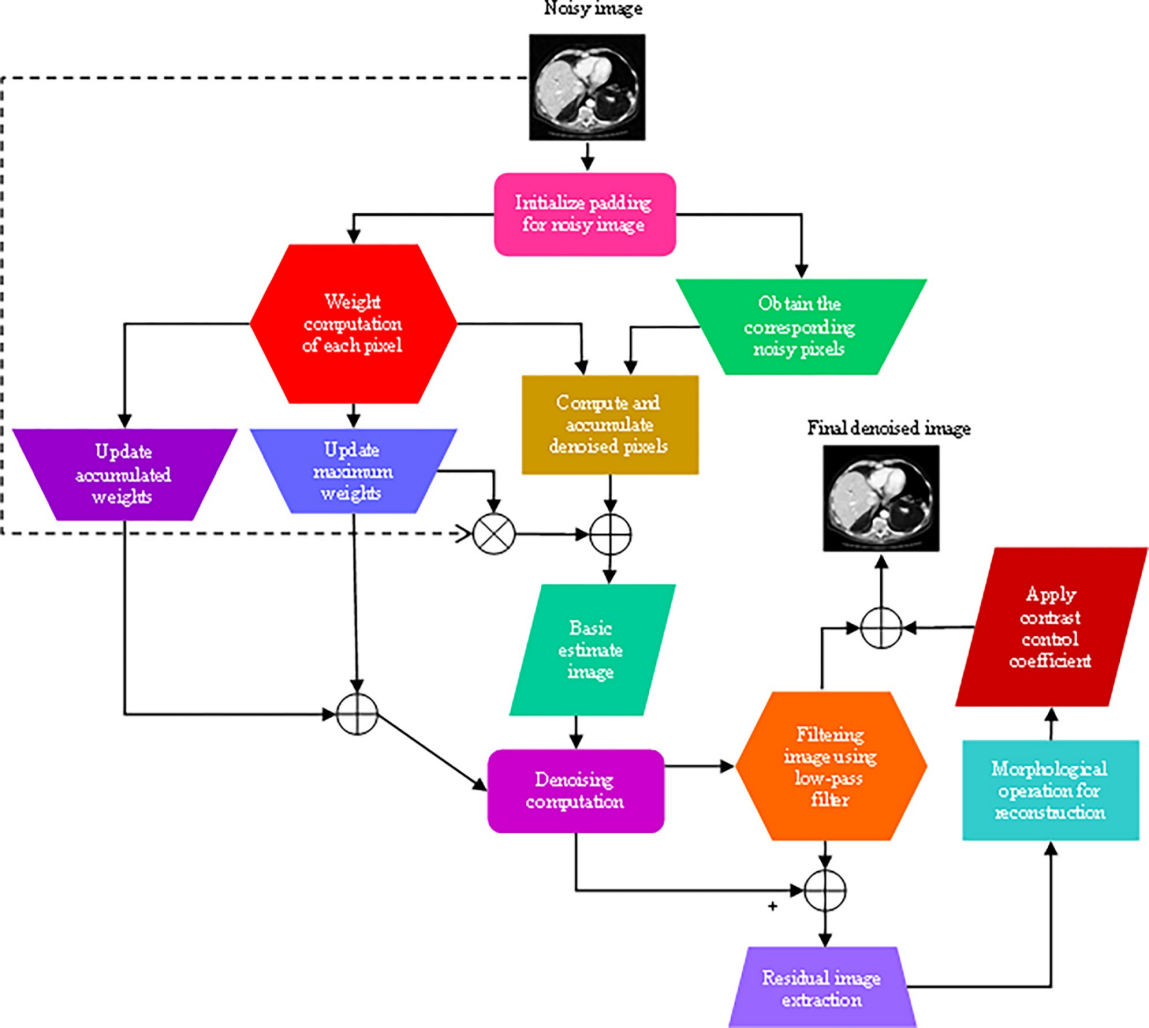
Flowchart of the proposed LDCT denoising algorithm.

This section put forward a detailed illustration of the proposed denoising framework. The flowchart of the proposed method is demonstrated in Fig 1. First, it employs efficient non-local mean filter that can deliver quality results. It constructs upon separable property of neighboring filtering to provide a rapid vectorized and parallel implementation in contemporary shared memory computer platforms. This strategy provides filters with a variety of desirable features as well as considerably reduces the noise and artefacts. Further, a morphologically processed residuals is used for edge preserving image processing. It combines linear lowpass filtering with nonlinear technique that enable to extract relevant image regions where edges could be preserved while removing noise and residual artefacts from the LDCT images.

### 2.1. Nonlocal means algorithm

A state-of-the-art algorithm was introduced to remove noise in CT scans with an assumption that CT images are noisy due to Gaussian noise. NL-means system [42] performs well when the image comprises abundant of repetitive structured patterns. It computes a weighted average pf pixel value by using redundant information to decrease noise. According to formal assumptions, images are assumed to be defined on the discrete regular grid Ω of size *d* and cardinality |Ω|. Let consider CT image be noisy and represented by *v*. The convex combination shows the value of recovered image *u* at the location s ϵ Ω as follows:

$$u(s)=\frac{1}{Z(s)}\sum_{t\in N(s)}w(s,t)v(t),$$ (1)

where N(s) is a pair of nearby locations of *s*, *w*(∙,∙) represents non negative weight and *Z*(*s*) is the normalization constant that provide us *Z*(*s*) = $\sum_{t\in N(s)}w(s,t)$ for any site *s*, respectively. *N*(∙) is denoted as a searching window in Buades et al. [42]. A weight *w* (*s*, *t*) is a measure of how similar two square patches positioned at locations *s* and *t*, respectively. The following is its definition as

$$w(s,t)=g_{h}\left(\sum_{\delta \epsilon \Delta }G_{\sigma }(\delta ){\left(v(s+\delta )-v(t+\delta )\right)}^{2}\right)$$ (2)

where Δ represent discrete patch area that contains neighboring locations that represent a Gaussian filter of variance σ^2^. The parameter *h* controls the degree of filtering. The *g*_*h*_ function is generally utilized in expressions such as $g_{h}(x)=\frac{1}{1+(x^{2}/h^{2})}$ and $g_{h}(x)=e^{-x^{2}/h^{2}}$, where the latter of which is used in [42]. The former is used in our algorithm since it performs highly effective implementation. The nonlocal means reconstructs an image through computing the weighted average of pixel values that considers similarity in intensity and spatial properties of pixels. The equal-sized patches are used to measure similarity because they accurately represent regional structures around the locations in consideration. It is important to notice that pixel outside of N(s) does not contribute the value of *u*(*s*). In the domain decomposition scheme, this property permits us to split an image into separate, unconnected parts and progress them in parallel.

According to [42], we adopt searching window N and patch *Δ* maintains the uniform cardinalities. The Eqs 1 and 2 are used to implement non-local means algorithm [42] that has *O*(|Ω|*K*^*d*^*P*^*d*^) for time complexity. Be careful because this complexity has an exponential with spatial dimension *d* but polynomial with number of pixels. The method continuous to be polynomial when dimensions are fixed and less in practice. To make the average more resilient, one could want to make the *N*(∙) as large as possible and finally stretch it throughout the entire image. However, this led to an unreasonable lengthy computation time, thus we limit our search to the closest neighborhood as recommended in [42]. We recommend the readers to [43] for the different definition of neighborhood filtering along the contexts of non-local means.

### 2.2. A constructive non-local means algorithm

The fundamental concept of the proposed strategy is to precisely calculate the weights *w*(*s*, *t*) provided by the Eq 2. When producing the restored image *u*, the weight computation takes the longest time. We present a simple tactic to making 1-dimensional images more understandable, which can be easily extended to large sides. Under the 1-dimensional hypothesis, we have Ω = ⟦0, *n*−1⟧ for the images having *n* pixels. Based on the specified translation vector *d*_*x*_, we present an image $S_{d_{x}}$ as denoted by

$$S_{d_{x}}(p)=\sum_{k=0}^{p}{\left(v(k)-v(k+d_{x})\right)}^{2},\;p\in \Omega$$ (3)

where $S_{d_{x}}$ represent discrete amalgamation of the squared difference between an image *v* and its translation by *d*_*x*_. Remember that Eq 3 is need access to the pixels outside the image domain. In order to restrict corruption of cache, we symmetrically or periodically expand the image boundaries. Remember that in 1-dimensional, we have patch of the form *Δ* = ⟦−*P*, *P*⟧. We substitute a Gaussian filter with a constant without clear variations. Then, the Eq 2 is transformed as

$$w(s,t)=g_{h}\left(\sum_{\delta_{x}\in \Delta }{\left(v(s+\delta_{x})-v(t+\delta_{x})\right)}^{2}\right),$$ (4)

Let *d*_*x*_ = (*t*−*s*) and define $\hat{p}=s+\delta_{x}$. With this reparameterization enables us to write as

$$w(s,t)=g_{h}\left(\sum_{\hat{p}=s-P}^{s+P}\sum_{\delta_{x}\in \Delta }{\left(v(\hat{p})-v(\hat{p}+d_{x})\right)}^{2}\right),$$ (5)

By dividing sum and further applying identity in Eq 3, we can define as

$$w(s,t)=g_{h}\left(S_{d_{x}}(s+P)-S_{d_{x}}(s-P)\right)$$ (6)

where in the fact independent of *t* given by quantity $S_{d_{x}}$ is known. A critical expression which permits us to calculate the weight for the set of in a given length of time. In general, for large sizes correspond to have combinations with image orthogonal axis in Eq 3. This method unquestionably results in a weight computation formula that calls for *O*(2^*d*^) operations on d-dimensional images. The formulation in [42], where it calls for operations for each patch, does not account for this method which is unaffected by the size of the patches. In short, the approach basically functions as follows: First, all values $S_{d_{x}}$ are calculated utilizing Eq 3 and the weights are determined utilizing Eqs 2 and 4. Then the denoising computation is executed utilizing Eq 1. The process is repeated for all probable translation provided by the dimensions of searching window.

### 2.3. Morphological residual processing

This section illustrates morphological residual processing [44] in detail. It is mainly based on the residual of a Gaussian filter and illustrate as follows.

$$I_{o}=u*L$$ (7)

where * is a convolutional operator and *u* is the denoised output of nonlocal means from Eq 1. ℒ is the Gaussian filter or any other lowpass filter mask. Henceforth, a linear filter residual is defined as

$$Res(u)=u-I_{o}$$ (8)

The residual is then put through another processing utilizing functions that are described using positive value images. In that case, the *Res*(*u*) that include both positive and negative values is decomposed into two parts ~ positive and negative.


$$I_{res}+=0.5(Res(u)+|Res(u)|);$$

$$I_{res}-=0.5(|Res(u)|-Res(u))$$ (9)

The residual fraction follows the apparently related relationship described below:

$$Res(u)=(I_{res}+)+(I_{res}-)$$ (10)

Both fractions of the residual (*I*_*res*_+, *I*_*res*_−) are processed to cutoff an irrelevant dissimilarity of the residual while maintaining relevant ones. The processing method which selects most significant residuum regions and preserves their original structures is based on morphological function ℳ and reconstruction. Lastly, significant residual areas are added in the image for recovering meaningful edges while simultaneously images remain blurs across irreverent image regions as

$$I_{out}=I_{o}+M(I_{res+})-M(I_{res-})$$ (11)

An operator ℳ, similar for both residual is described by

$$M(I)=R_{I}\left(min(I,S_{t}(I){|}_{\left\{min\{I\},max\{I\}\right\}})\right)$$ (12)

When *R*_*I*_ (*A*) represent morphological restoration of gray level mask image *I* from the marker *A* and ‘|’ is a mapping operator where an image is recovered by transforming a binary image by substituting primary values of 0’s and 1’s by two specified ones. A ′*min*′ is a pointwise minimal function of two images. A binary masker *S* that consists of significant regions where contrast can be maintained is the key aspect for the interpretation of ℳ. The substitute of these regions is based on the amplitude of residuum as

$$S_{t}(I)=(I\geq t)$$ (13)

where *S* represent selection function that extract regions of *I* whose amplitude exceeds a specific threshold *t*. The number of regions where the original sharpness of regions is recovered.

#### 2.3.1. Size criterion for selection of meaningful regions

According to initial implementation of this strategy, the edges of relevant areas are present in the binary mask that is extracted via thresholding. Using the amplitude residual, a meaningfulness is calculated. So far, amplitude is not the only factor that affects how significant an image component. It is easy to envision an image component with a greater residual amplitude specification that has no impact on how the image should be perceived. For instance, addition of salt and pepper to the image would result in the generation of several smaller image components with larger amplitudes and the addition of high amplitude components, which would alter the original image. Subsequently, they could be detected as relevant parts that is not optimal. Thus, further another set is incorporated to the original approach to tackle with this problem. Binary mask is filters by the area opening filter [45]. The binary image is removed by this filter, all related components with the size smaller than given threshold *s* (size coefficient). Subsequently, Eq 13 is expressed as

$$S_{t,s}(I)=(I\geq t)o(s)$$ (14)

where *o*(*s*) represents an area opening which remove elements smaller than that of specific size specified by *s*. As compared to initial method, one can notice that in both situations, when the coefficient (*s* in terms of modified method and *t* in terms of initial method) enhances, number of selected regions reduces. In contrary, in case of original situation, when the coefficient *s* is employed, the parts are eliminated related to their sizes. It permits thus for rejecting smaller, in case of number of pixel and objects from residuals even if their amplitudes are higher. Finally, it offers to keep these parts blurred on the output image.

#### 2.3.2. Control contrast

Addition (subtraction ~ depending of the components) of high-pass filtering from original image is one of the most well-known methods for improving contrast in images. It has to do with high pass filtering property to distinguish local differences in value of image pixels. Additional approach to get high pass filter results is to compared differences across lowpass filtering and image itself. High frequency components of images with amplitude areas exceeding threshold *t* are referred to as morphologically processed residuals. In order to control the contrast in the final denoised image, a contrast control coefficient (*c*) is introduced in Eq 11, which provides the updated formula as

$$I_{out}=I_{o}+(M(I_{res+})-M(I_{res-})).c.$$ (15)

By depending on *c*, can be further enhanced, preserved and reduced. Also shown are the results of contrast enhancement without morphological processing of its residual. How morphological processing affects this information can be realized in the quantity of small image features that is visible in the output [44]. Contrast of the relevant regions is improved by this processing method since it is enables to eliminate information that is unimportant. This strategy is controlled by the mainly three factors *t*, *s*, and *c*. The amount of information obtained depends on amplitude of residual (*t*) and particle size of the residual (*s*). The parameter *c* allows contrast of an output image to be controlled. Fig 9 determines the performance of our method in case of amplitude of residual (*t*).

## 3. Experimental results and discussion

This section presents visual and quantitative comparisons between the proposed algorithm and other comparative techniques including modified block matching 3D filtering (*m*- BM3D) [19], residual convolutional network with fractional TV loss (RCN-FTVL) [29], image decomposition and sparse representation (IDSR) [20], dilated residual convolutional neural networks (DRCNN) [34] and probabilistic self-learning framework (PSL) [46]. The peak signal-to-noise ratio (PSNR), feature similarity (FSIM), structural similarity (SSIM) and root mean square error (RMSE) are four quality indicators that are used to objectively evaluate the effectiveness of the given methods [47]. The intensity difference between a source image and the denoised image is calculated by PSNR. However, PSNR and RMSE do not authenticate image visual quality which is indeed significant for the justification of medical images. In comparison, SSIM and FSIM better expresses feature resemblance between denoised images and the reference image which helps to describe important information.

### 3.1. Dataset

The 2016 AAPM Mayo Clinic Low-Dose CT Grand Challenge dataset is utilized for this study [48]. This database contains both standard and low dose CT images of 1mm and 3mm thickness obtained from ten anonymous patients. This study considered only 3mm CT images. The dataset contains scans of chest, abdominal and head images from a multidetector row Siemen 16 CT scanner with 120 kVp tube voltage. Low-dose images are composed of the reduced tube current of 30 mAs compared to normal dose images which use a higher tube current of 240 mAs. However, in our study, the corresponding low-dose images are generated by adding Gaussian noise into the sinograms simulated from the NDCT images. The Gaussian noise is added to all of the images at different intensities with sigma values ranging from 30 to 5. This will allow us to simulate different doses and estimate the results of the proposed technique while also controlling the noise levels in the data. The severe streak noise and artifacts are visible in simulated LDCT images especially in local proximity to tissues with high attenuation components such as bones. Patient L035 data (abdominal CT scan), L129 data (abdominal CT scan), and L250 data (abdominal CT scan) datasets are considered in our experiments for evaluation. The NDCT images are considered as the ground truths for estimating the quality indices.

### 3.2. Parameter settings

In case of existing LDCT image denoising techniques (*m*-BM3D, PSL, IDSR, DRCNN, and RCN-FTVL), parameters are set as instructed by the authors of the respective articles. In terms of proposed algorithm, the parameters *P* in Eq 6 are generally set to 5, while parameters *K* and *h* were set to 3 and 0.15, respectively. Also, the values of parameters such as the size of residual particles (*s*) and the amplitude of the residual (*t*) typically rely on the level of information that is intended to be controlled. The parameter (*c*) controls contrast of the denoised results. We set t = 0.15, s = 3, and c = 1.2 for all datasets in our method. All the experiments are implemented on CPU Intel (R) Core (TM) i9-9900K CPU @3.60GHz with 8GB RAM using MATLAB.

### 3.3. Qualitative analysis

**Fig 2:**
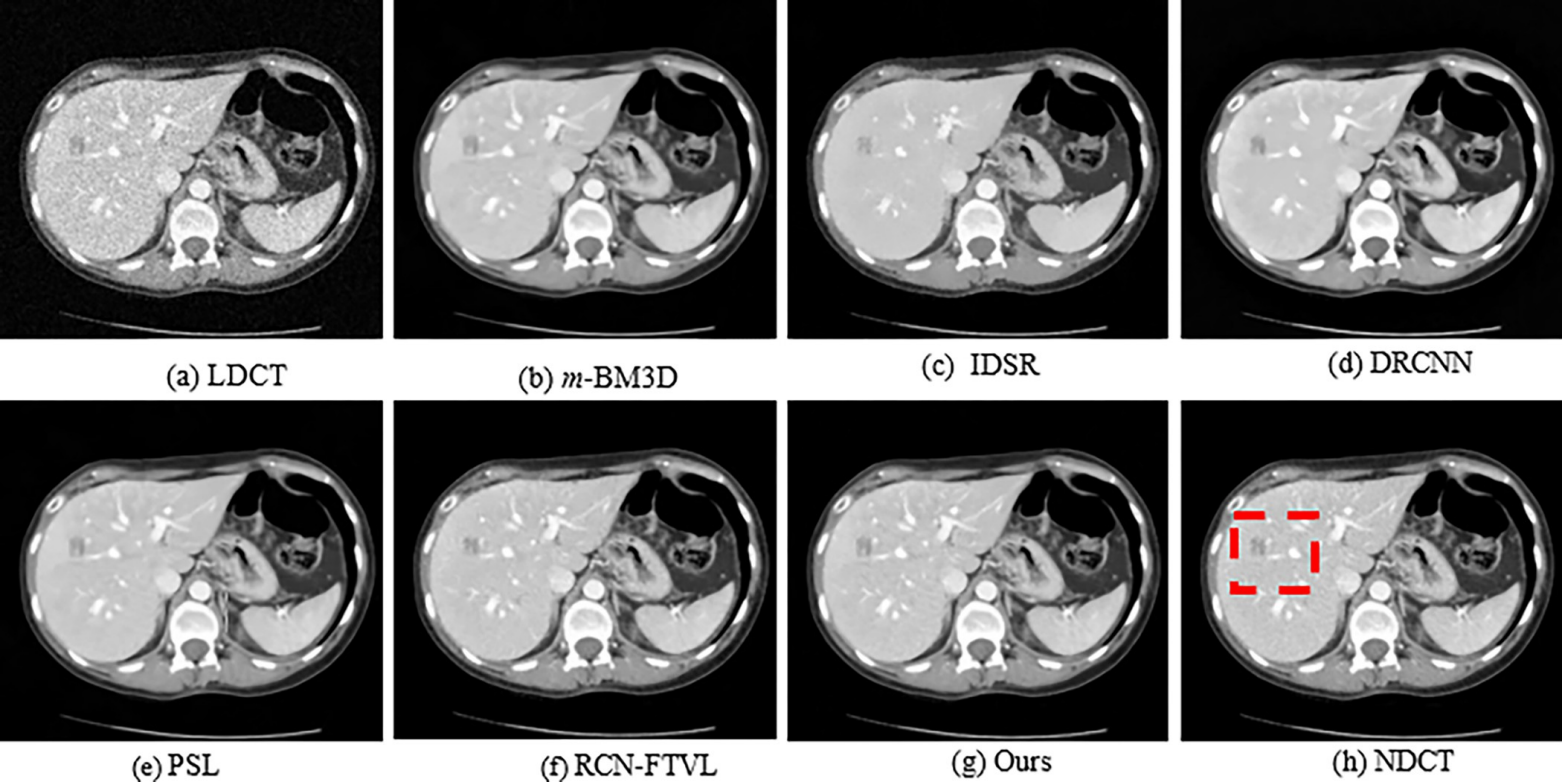
Qualitative results of numerous methods using L035 dataset. A red rectangle designates the regions of interest (ROI). The display window makes it simpler to visualize the lesion.

**Fig 3:**
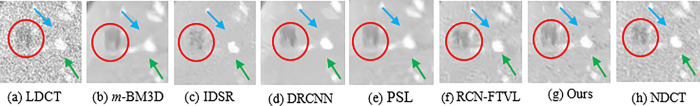
The regions of interest (ROI) are shown for better comparisons by red circle from Fig 2. The two subtle structural regions are pointed by green and blue arrows.

**Fig 4:**
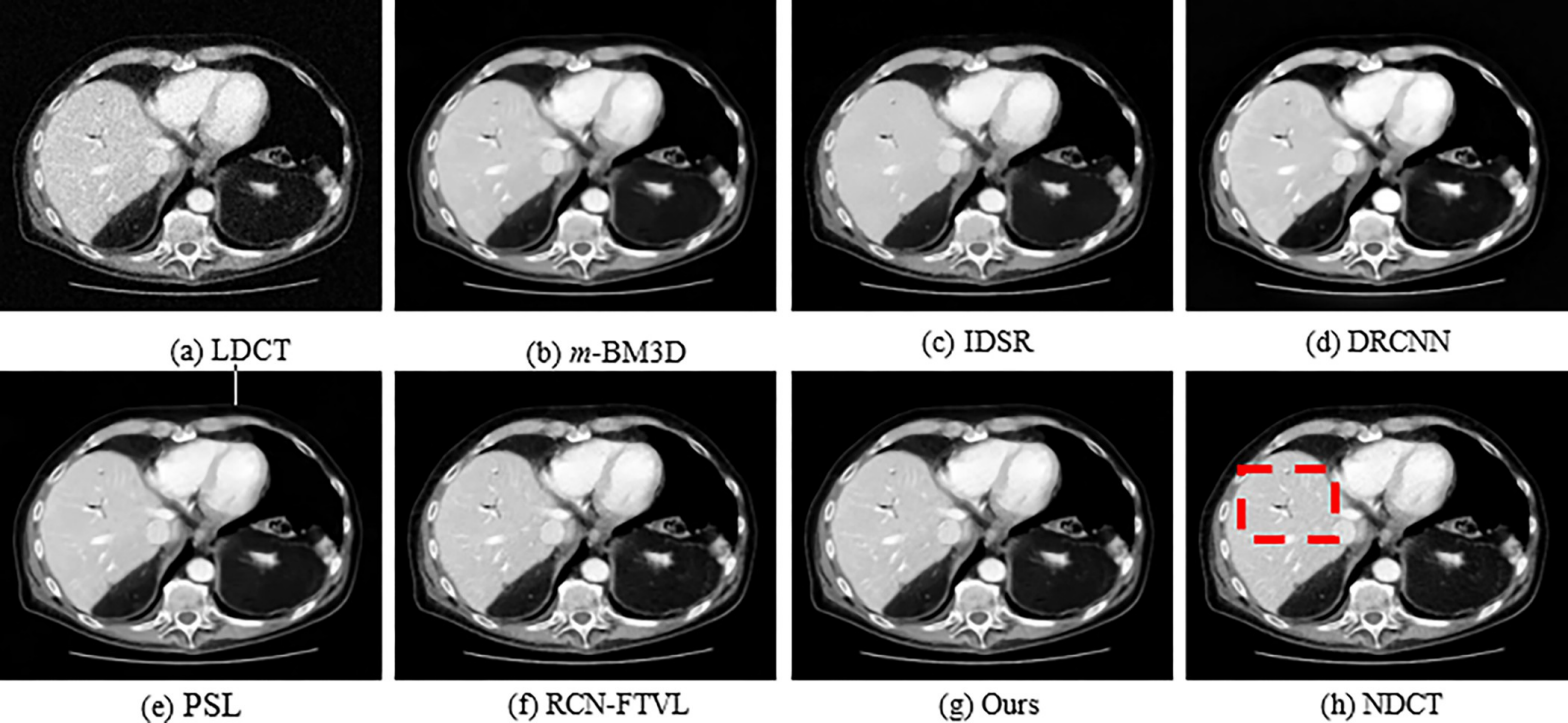
Qualitative results of various methods using the L129 dataset. A red rectangle designates the regions of interest (ROI). The display window makes it simpler to visualize the lesion.

**Fig 5:**
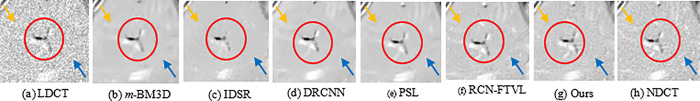
The regions of interest (ROI) are shown for better comparisons by red circle from Fig 4. The two subtle structural regions are pointed by orange and dark-blue arrows.

**Fig 6:**
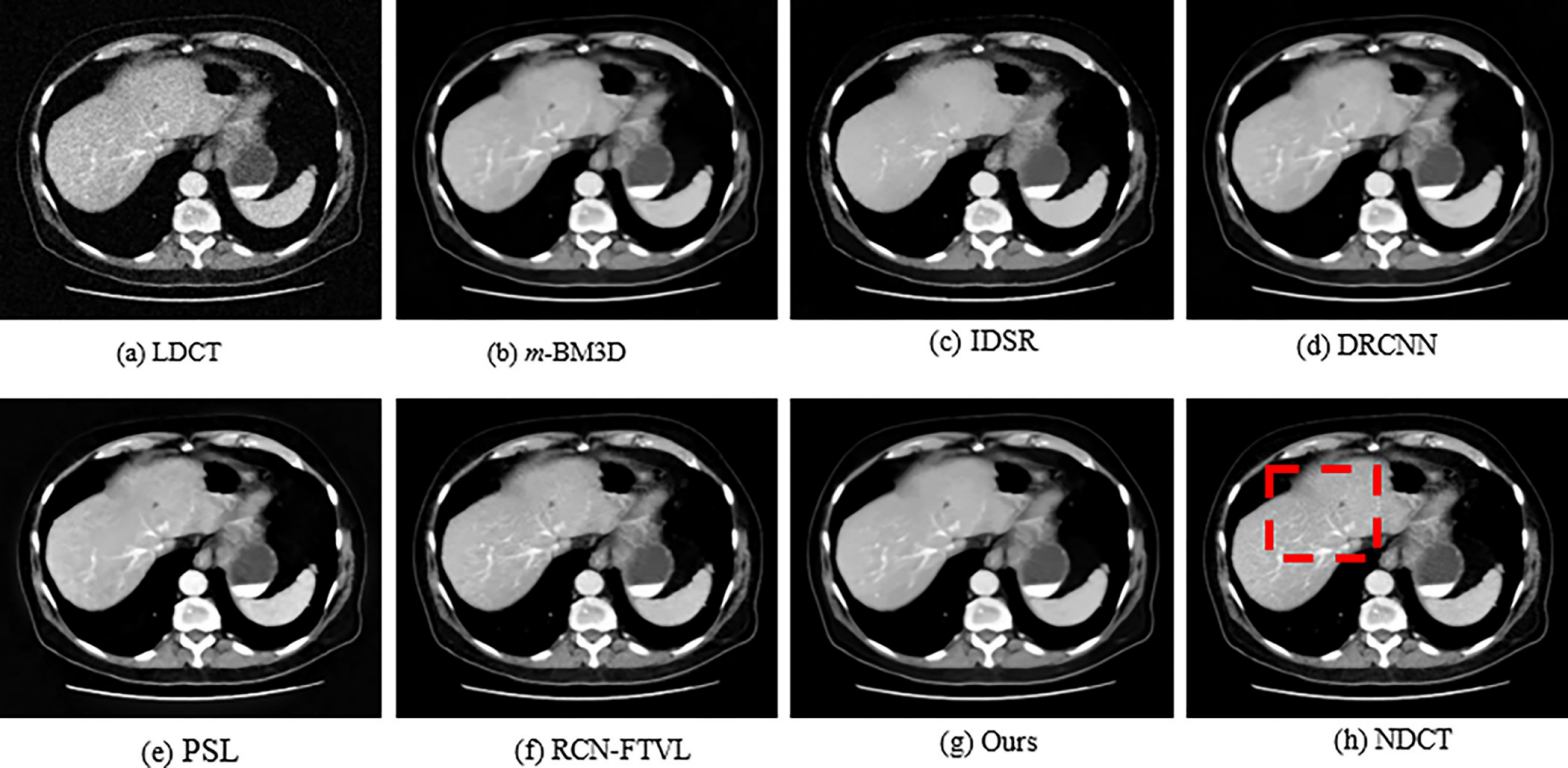
Qualitative results of various methods using the L250 dataset. A red rectangle designates the regions of interest (ROI). The display window makes it simpler to visualize the lesion.

**Fig 7:**
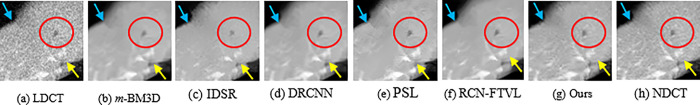
The regions of interest (ROI) are shown for better comparisons by red circle from Fig 6. The two subtle structural regions are pointed by the yellow and blue arrows.

In order to qualitatively evaluate the denoising results, three set of CT slices that represent an abdominal region are considered from the dataset and exhibited with denoising results using several techniques in Figs 2, 4 and 6, respectively. Figs 2H, 4H and 6H observed NDCT images of several features with sharp edges, tissues as well as low density lesions. However, the corresponding noisy images shown in Figs 2A, 4A and 6A with visual degradations. The experimental results of a simulated abdominal CT scans L035, L129 and L250 considered for analysis. The denoising results of *m*-BM3D, IDSR, DRCNN, PSL, RCN-FTVL, and the proposed method are demonstrated in Figs 2B–2G. In Fig 3, red circles represent lesions. By comparison, the *m*-BM3D approach is effectual for denoising LDCT images but it could not obtain better results in noise removal task and fails to preserve details in optimal level. In Fig 3B, for example, region of interests (ROIs) is more smooth and vaguely visible. IDSR outperforms *m*-BM3D in terms of maintaining features, however as observed in Fig 2C, there is a blocky effect in the edge regions. The DRCNN shows better performance while maintaining details and edges as shown in Fig 2D, where the edges and textural information noticeable and generates ideal contrast. But DRCNN is inefficient in flat areas particularly in heavily contaminated regions. Note that, DRCNN needs numerous iterations to improve performance as the denoising results. The regions of interests are highlighted in red circles for clear observation in Fig 3. In Fig 4, it can be seen that m-BM3D and IDSR efficiently suppress mottle noise and streak artefacts however the edges and the details are blurred. DRCNN has lost texture information as shown by the arrow in Fig 5D. However, it is abundantly clear from the visual representations that the structural and textural features of DRCNN have been improved by the deep learning-based methodology. However, some soft tissue boundaries have been smoothed by the PSL and RCN-FTVL in those smooth areas has led to streaking artefacts. Figs 5 and 7 zoomed in one region of interests (ROI) represented in Figs 4 and 6, respectively to further highlight noise reduction and detail retention of the specific images. The ROIs of L129, which are shown in Fig 5 display a solid non-calcified lesion which is denoted by orange and blue arrows. This lesion region has been effectively retained by the majority of algorithms. According to the DRCNN result in Fig 6D, the visibility of the bone structures and lesions has marginally improved. But the edges still haven’t fully recovered their sharpness. The image has poor texture preservation when it is visualized. From the Fig 6E and 6F denote how the performance from PSL and RCN-FTVL techniques successfully preserved the lesion and other structural characteristics. When it comes to texture preservation and artefact reduction, the proposed method outperformed PSL and RCN-FTVL results. A metastasis is highlighted by the ROIs represented in in Fig 7 of the abdomen CT image from Fig 6. Due to the impact of noise, the lesion is not evidently visible in ROI of LDCT demonstrated in Fig 7A. The metastatic region is oversmoothed in the m-BM3D and IDSR presented in Fig 7B and 7C, even if the chosen denoising techniques have somewhat decreased noise in every of these sub-images. The lesion region is shown in the DRCNN with blurry edges. Furthermore, streaking artefacts have interfered with smooth portions of PSL presented in Fig 7E. In addition, compared to the RCN-FTVL result shown in Fig 7F, our proposed technique as shown in Fig 7G has maintained textural and structural information considerably sharper. In the flat regions as shown in Fig 7D, it works well in terms of maintaining details but in the curved regions, it works poorly. RCN-FTVL in Fig 7F executes well in removing noise and artefacts but the edges are somewhat blurred. Therefore, it could be described that the proposed method could denoise LDCT images reasonably well than the other methods.

### 3.4. Quantitative analysis

Tables 1 and 2 demonstrates quantitative results of PSNR, SSIM, FSIM, and RSME obtained from different algorithms for all the datasets. The ideal among all these algorithms is emphasized with bold. In Tables 1 and 2, it shows that our method generates comparatively ideal results for all the test samples. The values for all these metrics vary for different methods. The proposed technique achieved the lowest RMSE value for the test image, as observed by the findings in Table 1. A minimal RMSE indicates that the noise level in the proposed method has been significantly reduced. Similarly, this judgement is also constant with all the samples verified utilizing PSNR. The PSNR scores for the tested image samples show that m-BM3D has the lowest PSNR. According to the SSIM and FSIM scores tabulated in Tables 1 and 2, the PSL and RCN-FTVL can preserve structural features better than the other approaches. The results in Table 1 indicate that the RMSE values gradually decrease from the PSL, RCN-FTVL, to the proposed approach. This trend highlights the capability to decrease the noise. The highest value is shown in the proposed technique and continues to rise from PSL and RCN-FTVL among all other methods. The PSNR signifies the overall visual quality irrespective of the spatial information. The results provided in Table 1 indicate that PSNR obtained using proposed method outperforms all other approaches. Similar trend for SSIM, RMSE and FSIM was observed along with PSNR scores. The SSIM and FSIM of our algorithm is more than SSIM and FSIM of other techniques due to improved contrast. For all test images, the proposed approach produces higher PSNR, SSIM and FSIM values. The proposed strategy yields necessary best results across all test samples. To analysis, the graph-based assessment technique sums up, our technique obtains finer results in case of noise removal as well as preserving information when compares to other comparative algorithms. In addition to qualitative as well as quantitative of denoised image i.e., intensity profile has been implemented where it compares the line profile of denoised image with the reference image.

**Table 1:** Quantitative indices PSNR, FSIM and RMSE scores of different methods on L035, L129 and L250. The bold values represent the best results.

Method	L035			L129			L250		
	PSNR (↑)	FSIM (↑)	RMSE (↓)	PSNR (↑)	FSIM (↑)	RMSE (↓)	PSNR (↑)	FSIM (↑)	RMSE (↓)
**LDCT**	24.6827	0.9355	0.0487	24.0722	0.9411	0.0535	23.2526	0.9211	0.0545
*m*-BM3D [19]	26.8262	0.9588	0.0436	25.3552	0.9542	0.0520	24.9922	0.9324	0.0522
IDSR [20]	27.2524	0.9614	0.0422	25.8252	0.9623	0.0513	25.4424	0.9672	0.0509
DRCNN [34]	27.5927	0.9628	0.0419	26.5662	0.9732	0.0497	25.7725	0.9712	0.0492
PSL [46]	27.9626	0.9713	0.0413	26.9524	0.9752	0.0475	26.6722	0.9784	0.0473
RCN-FTVL [29]	28.1828	0.9784	0.0402	27.3827	0.9887	0.0463	26.9926	0.9887	0.0457
**Ours**	**28.5610**	**0.9895**	**0.0389**	**27.8297**	**0.9926**	**0.0447**	**27.6327**	**0.9911**	**0.0435**

**Table 2:** Quantitative indices SSIM scores of different methods on L035, L129 and L250. The bold values represent the best results.

Method	Dataset		
	L035	L129	L250
LDCT	0.7617	0.7226	0.7828
**m**-BM3D [19]	0.7915	0.7683	0.8026
IDSR [20]	0.8015	0.7762	0.8193
DRCNN [34]	0.8266	0.7925	0.8263
PSL [46]	0.8516	0.8262	0.8366
RCN-FTVL [29]	0.8715	0.8285	0.8526
Ours	**0.8816**	**0.8546**	**0.8627**

**Fig 8:**
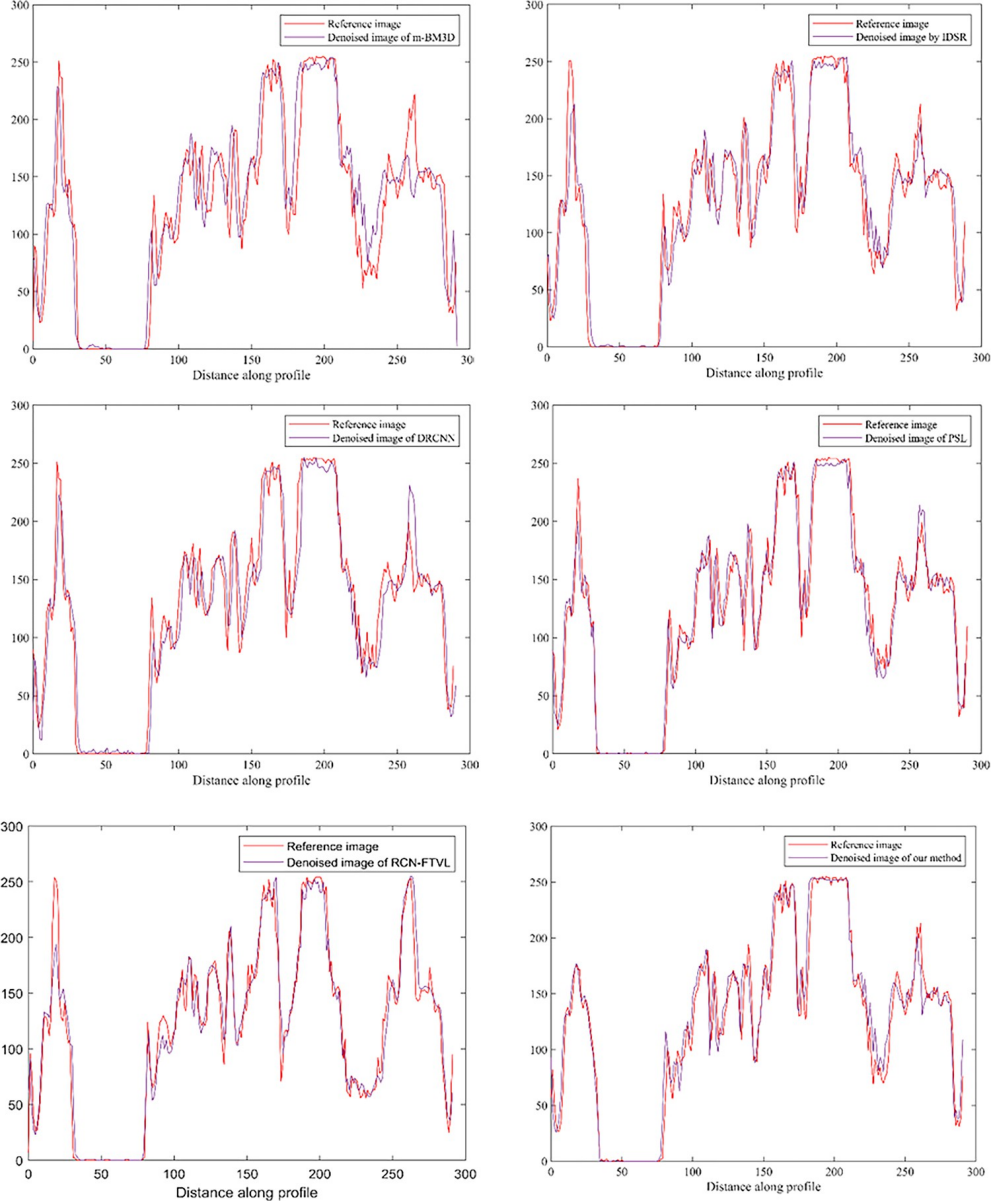
Intensity profile of reference image (L035) against the denoised images of m-BM3D [19], IDSR [20], DRCNN [34], PSL [46], RCN-FTVL [29] and the proposed method, respectively.

The intensity profile is a line graph that plots the pixel values along the line segment of an image. If the intensity profile of line segment of denoised image overlaps the intensity profile of line segment of reference image, then the denoised image is good. However, if the intensity profile of denoised image does not overlap, then the denoising results are not good. Fig 8 denotes the overlapping scenario of intensity profiles of reference and denoised images. From the Fig 8, it clearly presents that the overlapping performance of our algorithm is better compared to other comparative algorithms and it concludes that denoising efficiency of our algorithm is satisfactory comparing to other algorithms.

## 4. Ablation study

In this section, comparative experiments are conducted to study the impact of the constructive non-local means model, morphological residual processing and the size criterion approach.

### A constructive non-local means model

This model allows for the decomposition of an image into disjoint parts, allowing for parallel denoising of the distinct image parts. This fact has already been researched in order to enhance computation times. However, combining image decomposition with this technique enabling vectorization of operations using contemporary SIMD (Single Instruction Multiple Data) instructions set results in a significantly faster speed improvement. This is equivalent to parallelism at the data level. To use SIMD instructions, our solution must strictly adhere to the constraint of accessing aligned data in memory, which is not required when implementing non-local means with sliding windows. Our method makes use of the most recent SIMD instructions set and makes use of the prefix sum construct to effectively build images in a cache-aware manner. It is crucial to note that, while using SIMD instructions and maximizing cache hits do not improve the temporal complexity of an algorithm in the RAM model, they do significantly reduce the computational load of its implementation.

### Morphological residual processing

It works on the principle of extracting the low-pass linear filter residuals, which are then processed using a non-linear technique based on morphological operators of area opening and reconstruction. The output of the later processing is added back to the result of the low-pass filtering. Non-linear filtering attempts to localize and extract relevant edges restored on the final image such that blurring does not occur inside their regions. The method’s behavior is determined by four parameters: the mask of the Gaussian (or other low-pass linear) filter, the threshold *t*, the size coefficient *s*, and the contrast coefficient *c*. The final result may be adapted to specific requirements depending on the parameters selected.

### Size criterion approach

The amplitude of residuals is used to estimate meaningfulness. However, amplitude is not the only criterion that affects the relevance of an image region. Image elements with a high amplitude of residual are easily characterized as being unimportant for image understanding. For example, the addition of salt-and-pepper noise to the original image generates a large number of tiny (one-pixel-size) image elements of high amplitude on the input image and consequently alters the residual image by adding high-amplitude elements. As a result, they would be identified as meaningful regions, which is undesirable. To address this issue, an additional step is added where an area opening filter is used to filter the binary mask. This filter removes from the binary image, all connected components with sizes smaller than a specified size threshold *s* (size coefficient).

## 5. Discussion

The study presents a significance of LDCT image denoising that can reduce undesired noise and streak artefact, which degrades the diagnostic performance. For analysis, we used a number of images from the standard dataset [48]. Both NDCT and simulated LDCT images of the corresponding images are presented in Figs 2–7. The regions of interest (ROI) of local regions represented by red boxes and red circles are shown in Figs 2–7 in case of accurately comparation of resultant images of numerous strategies. The LDCT images clearly show streak artefacts and mottling noise. We can see that the proposed strategy significantly improves image quality while effectively suppressing streak artefacts. The reconstructed images by *m*-BM3D algorithm shows that there are still some artifacts and noise. From the overall visual analysis, the images obtained IDSR suffer from noticeable blocky effects. The results obtained from DRCNN method, PSL method, and RCN-FTVL method still have a tendency to generate residual artifacts.

**Fig 9:**
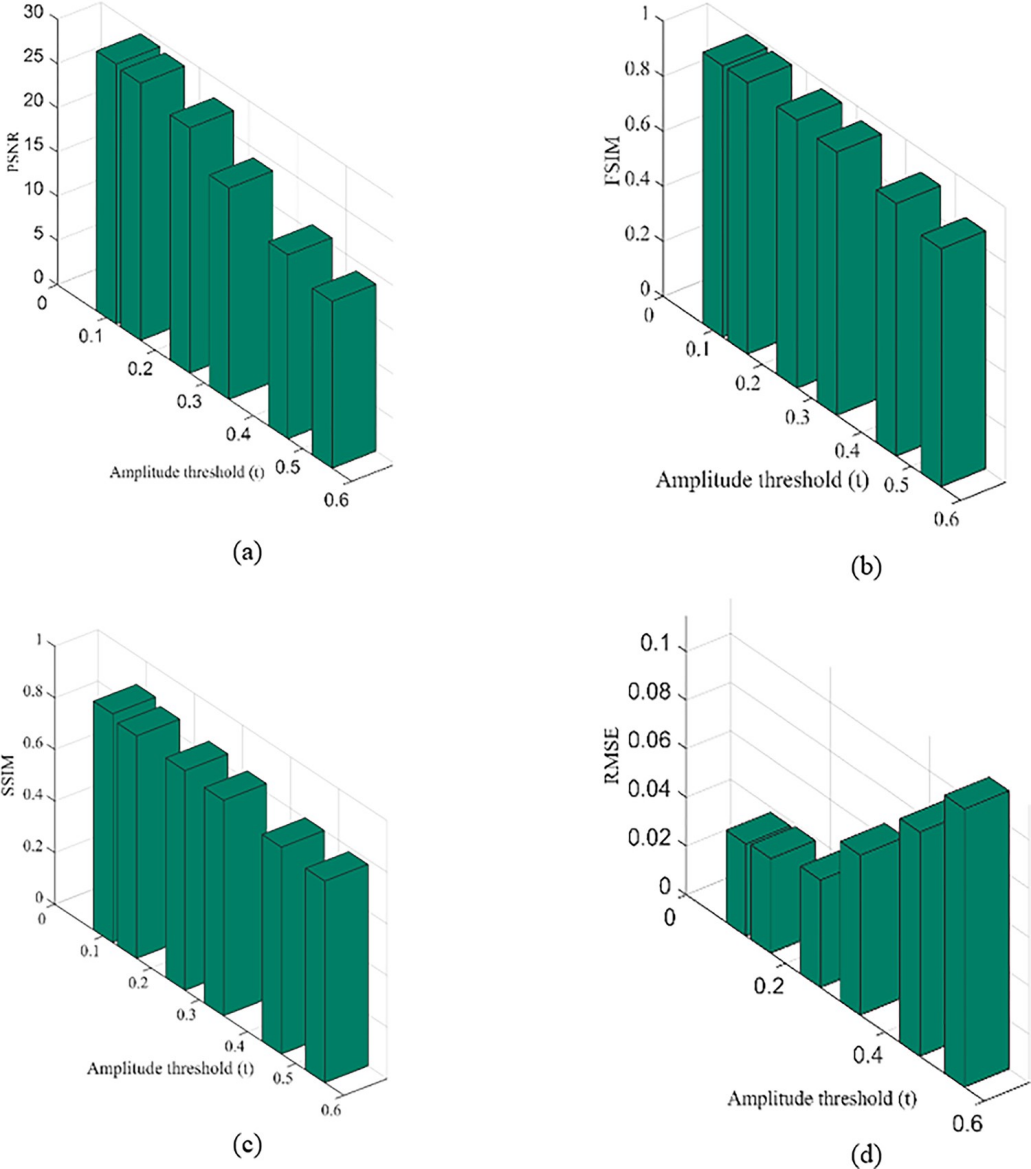
The influence of amplitude threshold (*t*) on the proposed method with respects to PSNR, SSIM, FSIM and RMSE.

**Fig 10:**
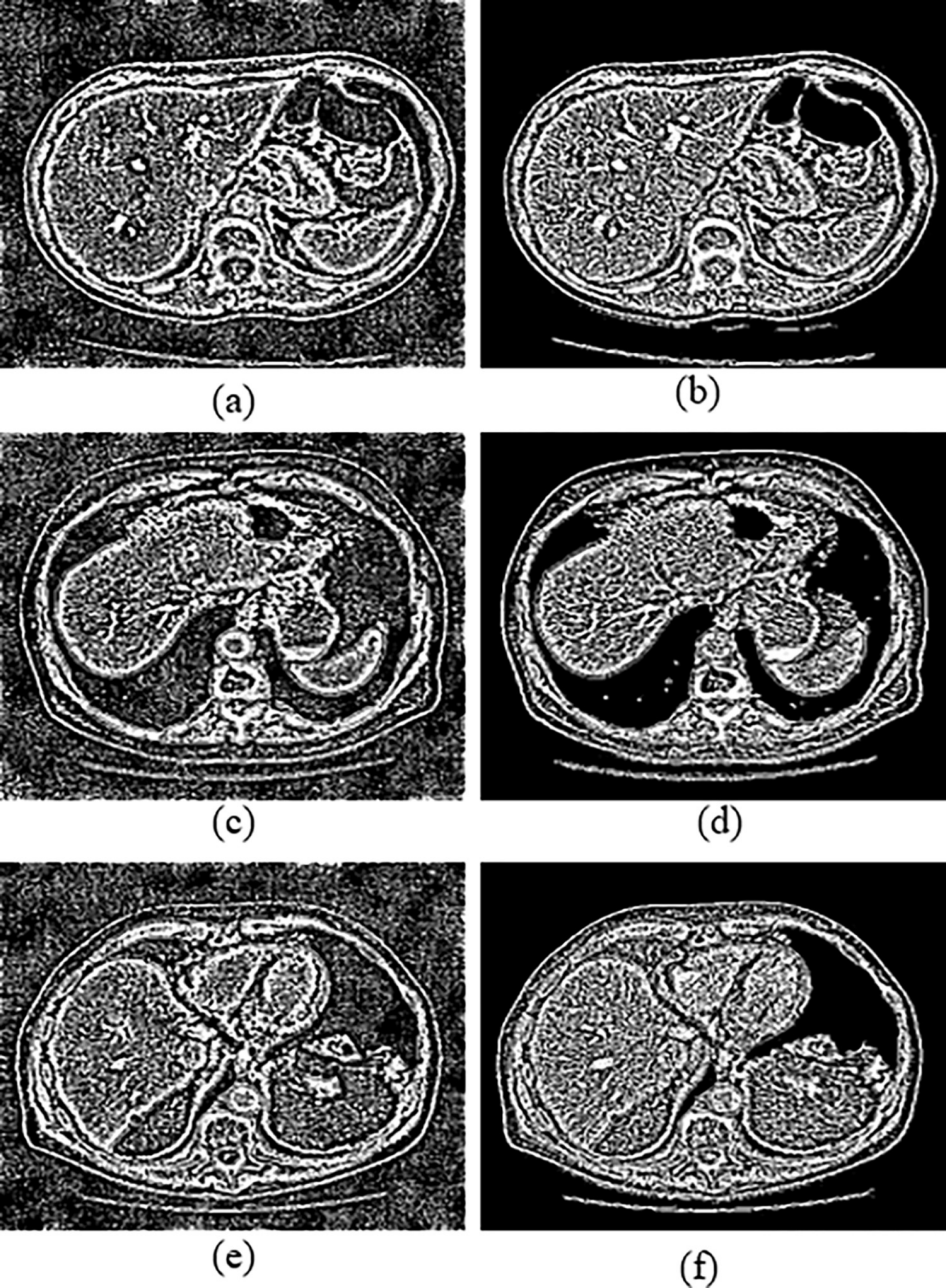
Residual images: (a, c, e) represents low-dose CT images and (b, d, f) represents corresponding denoised images of the proposed method on L035, L129 and L250.

Although the PSL denoising result is finer than DRCNN, it could not totally suppress noise, but performs best in flat regions than DRCNN. Fig 9 shows that PSNR, SSIM, FSIM and RMSE scores of proposed method in terms of amplitude threshold (t). The highlight of Fig 9 is that the PSNR, SSIM and FSIM of our method is higher than other methods, however its RMSE score remain less which is ideal for better performance. The score of the proposed algorithm always exhibits better values when compares to *m*-BM3D, IDSR, DRCNN, PSL and RCN-FTVL. We conclude that our method has generated superior performance efficiency to its competitors. RCN-FTVL algorithm suffers from residual noise. Noise and artefacts have been eliminated while maintaining structural details impressively by the proposed method. Overall, our method outperforms others when it comes to denoising performance. In all cases, our method yields higher SSIM or FSIM values and lowest RMSE. Further, the noise in region of interest (ROIs) is substantially lowest in our method and obtains somewhat appropriate than other algorithms in all the ROIs. The original LDCT images clearly shows mottle noise and streak artefacts. The *m*-BM3D denoising method performs well at removing noise and artefacts but the blocky effects are noticeable especially in flat areas. The regions in the denoised image obtains using IDSR approach still suffers by mottle noise however it works pretty well in case of preserving edges and features. However, there are still plenty of lots of residual streak artefacts visible. In comparison to other competing methods, our method is the typically effective. The PSNR, SSIM, FSIM and RMSE are utilized to objectively evaluate the quality of images of denoised clinical CT scan images. It could be observed that the PSNR, SSIM and FSIM scores of our approach are highest while RMSE values of our method are lowest which indicates better performance. The quantitative evaluation criteria PSNR, SSIM, FSIM and RMSE tabulated in Tables 1 and 2 represents that our method demonstrates ideal results compares to other comparative approaches. Also, the denoised images demonstrate that proposed approach surpasses all other competing algorithms in case of visual analysis. That is to say, both a quantitative study and a visual result indicate to the accomplishment of the proposed strategy. Table 3 presents the computational time for execution of all methods (i.e., *m*-BM3D, IDSR, DRCNN, PSL, RCN-FTVL and the proposed method). We can see that the DRCNN and RCN-FTVL methods have the longest running times. This is so because deep learning models takes longer processing time during training and testing phase. Other approaches take less time besides these two models. As a result, our method requires much less running time for all of the test images when compared to the other competing methods. In addition, we can observe from the residual images represented in Fig 10 that the residual images generated by the LDCT images are noisy and exhibits poor visualization, however, residual images produced by the proposed denoised images are clearer and free on noise. This further indicates that the proposed method is able to remove noise from the LDCT images and exhibits visually appealing results.

**Table 3:** Average running times for the different LDCT denoising algorithms.

Method	Average running time (s)
***m***-BM3D [19]	0.0341
IDSR [20]	0.0485
DRCNN [34]	0.0627
PSL [46]	0.0527
RCN-FTVL [29]	0.0667
Ours	0.0286

## 6. Conclusions and future work

This study presented a competent LDCT denoising technique based on a constructive non-local means algorithm with morphological residual processing for the purpose of removal of noise and simultaneously improving the image quality of LDCT images. We proposed a fast approach for computing non-local filtering and presented the results after denoising noisy LDCT images. The proposed method uses an efficient version of non-local means filter that can able to generate better results. The fast vectorized and parallel implementation in recent common memory computer platforms based on the latest single instruction multiple data (SIMD) instruction set is faster and scales fine with image dimension as well as core count. For edge-preserving image processing, we also use lowpass filtering combined with residual morphological processing. In order to achieve this, it combines linear lowpass filtering with the non-linear approach which enables the selection of important regions where edges can be effectively retained. Using morphological processing of the linear filer residuals, the regions are selected with a focus on recognising important regions with high amplitude and precise size. In terms of noise and artifact removal, our technique performs better than other algorithms. The proposed approach effectively preserves structural and textural features. The iteration times are significantly reduced by the proposed method which makes a substantial contribution to the reduction of processing time and cost. However, the removal of noise from the LDCT images is still a challenging process since it contains higher spatial variations and correlations. The performance of denoising will continue to improve as soon as the parameters in the proposed approach can be properly adjusted according to the noisy input images. Although the proposed method produces high-quality LDCT images, the diagnostic performance has not been investigated which is the limitation of the proposed method. The practicability of utilising simulated CT images is examined however the diagnostic performance was not accessed. In the future, more efforts will be formulated to translate our technique for other denoising problems.
